# Physicochemical Prediction of Metabolite Fragmentation in Tandem Mass Spectrometry

**DOI:** 10.5702/massspectrometry.A0066

**Published:** 2018-06-14

**Authors:** Wataru Tanaka, Masanori Arita

**Affiliations:** 1 Department of Genetics, SOKENDAI (The Graduate University for Advanced Studies); 2 National Institute of Genetics

**Keywords:** spectral prediction, theoretical spectral library, quantum chemistry, *in silico* fragmentation

## Abstract

Current bottleneck of comprehensive non-target metabolite identification is insufficient spectral library. Many research groups have tried to build a theoretical product ion spectral library independent of measurement condition or settings, but mechanisms of metabolite fragmentation are not fully clarified. To achieve the mechanistic prediction of metabolite fragmentation which covers a wide range of metabolites, we will discuss utilization of physicochemical calculation. We introduce bonding patterns, which include two bound atoms and chemical groups adjacent to the bond. Cleavage of each bonding pattern is simulated and its activation energy is precisely calculated with quantum chemistry and assigned on metabolites. By tracing low-energy bond cleavages, fragmentation of a dipeptide molecule is successfully predicted. Prediction on another metabolite requires some additional features to fully reproduce its experimentally observed product ions. Physicochemical calculation shows its promising ability to predict fragmentation pathways only from metabolite structures, while required improvements suggested by comparison between our prediction and standard spectra stored in database are also discussed. Moreover, to construct a prediction strategy which withstands the vast metabolite space, we have to build a comprehensive list of bonding patterns and their activation energy. As theoretically possible bonding patterns are huge in number, proper simplification of the patterns must be implemented. We will discuss how to achieve it in addition to the prediction results.

## INTRODUCTION

In metabolome analyses, tandem mass spectrometry (MS/MS) is a powerful tool to identify metabolites from biological samples. In MS/MS, metabolites are fragmented into their substructures by colliding with neutral gas, and the fragmentation pattern at each time slice is recorded as a product ion spectrum, composed of *m*/*z* of product ions and their relative intensities. Since product ion spectra are different for each metabolite structure, they are used as fingerprints to identify metabolites, especially to distinguish structurally isomeric metabolites. For the purpose of metabolite identification, many databases, either commercial or public domain, provide standard product ion spectra: NIST Tandem Mass Spectral Libraries,^1)^ MassBank,^2)^ METLIN,^3)^ GNPS,^4)^ and Human Metabolome Database (HMDB)^5)^ for example. The matching of the standard spectrum with an experimentally observed one is considered a prerequisite of the metabolite identification.

An apparent drawback of the library approach is that no database is comprehensive in a true sense: we cannot prepare product ion spectra for all possible metabolites. In the PubChem database for example, over 94 million structures, including synthetic ones, are registered. To obtain a standard product ion spectrum, we need to measure a pure, *i.e.*, commercially extracted, metabolite with an MS/MS platform one by one, and the measurement is a complicated and costly process. It is therefore technically difficult to prepare a spectral library of more than thousands of standard compounds.

To overcome this intrinsic limitation, many research groups have challenged to predict product ion spectra only from molecular structures. For phosphoglycerolipids, whose fragmentation pattern is well known, a theoretical library has been constructed by extrapolating *m*/*z* of their product ions and their intensities for possible structural variants combinatorially.^6)^ This approach, called lipidomics for glycerolipids, should be applicable for other structure classes. The library construction, however, seems much harder for small metabolites, especially for those containing heteroatoms such as oxygen and nitrogen.

One successful spectrum prediction was achieved by machine learning systems which learn fragmentation patterns from experimental spectra.^7,8)^ Other groups systematically classified fragmentation patterns in order to understand the mechanism of fragmentation in MS/MS.^9–11)^ Nevertheless, how fragmentation proceeds in MS/MS remains largely unclear and theoretical clues for more accurate prediction have been awaited.

In the present study, we focus on physicochemical properties of metabolites. Previously we have calculated bond cleavage energy and electron distributions of some metabolite structures by means of computational chemistry and clarified mechanisms of collision induced dissociation (CID) of their protonated molecules in electrospray ionization (ESI) MS/MS.

Hydrogen rearrangement along metabolite fragmentation was formulated in MS-FINDER program and utilized to predict precise mass of fragments.^12)^ Existence of fragment structures of reserpine(+), 2′-deoxycytidine 5′-diphosphate(−), and 3-indoxyl sulfate(−) predicted by MS-FINDER program were confirmed with energy calculation; here, (+) and (−) stand for positive- and negative-ion mode ESI, respectively. Especially, we elucidated characteristic behaviors of phosphorus and sulfur atoms, whose experimental evidences were not enough. In addition, our physicochemical calculation proved existence of fragment structures unexpected by the MS-FINDER program. Phosphocholine(+) and kaempferide(−) produced radical ions which violate the even-electron rule^13)^ implemented in MS-FINDER, but our calculation elucidated their electron structures allowing the radical ions to stably appear in experimental measurements. Fragmentation of isoproturon(+) followed by immediate charge transfer was also confirmed by our calculation.

Utilization of the physicochemical calculation to annotate an unknown metabolite structure was exemplified by sphingolipid identification.^14)^ Fragmentation pathways of isobaric β- and γ-hydroxylceramides(−), whose structures were different only about positions of a hydroxy group, were simulated with computational chemistry. The computed fragment structure from the β-hydroxyl ceramide was consistent with a structure-unknown experimental product ion spectrum. Therefore, the spectrum was successfully annotated as the β-hydroxyl ceramide. From this calculation, chemical groups adjacent to a cleaved bond were found influential to energetics in fragmentation. As only C–C bond cleavage was subjected in that study, we will discuss extensibility of the idea to heteroatoms by dealing with other metabolites containing rich heteroatoms.

Here, we extend these physicochemical approaches to predict fundamental cleavage sites in CID of protonated molecules caused by positive-ion mode ESI-MS/MS. Each bond composing a metabolite is assigned a “bonding pattern” (BP) including its neighborhood, *i.e.*, two atoms and chemical groups adjacent to the bond. Then activation energy of each BP is calculated with computational quantum chemistry. Prediction of fragmentation schemes is performed by finding the lowest activation energy, and its comparison with experimentally measured spectra are explained. Example subjects discussed in the present paper are glycylleucine dipeptide and ethyl argininate, which possess rich heteroatoms making their fragmentation complex. Since any molecular structure can be regarded as a composition of BPs, our method is applicable to a variety of metabolites in principle.

The present method focuses on the prediction of easy-to-cleave bonds in metabolite structures, and not their temporal aspects (kinetics). Minimum energies to cleave bonds are calculated to create theoretical product ion spectra, dependency on applied CID energy, and other features. It is well known that chemical kinetics is also important, but our prediction method focuses on the usability of energy calculations to predict fragmentation schemes.

## EXPERIMENTAL

### Assignment of Bonding Patterns

BPs consist of two bound atoms and their adjacent chemical groups. They are assigned for all single bonds between a carbon atom and a heteroatom (nitrogen and oxygen), which are principal candidates for cleavage. Single bonds between two carbon atoms or multiple bonds are excluded from the cleavage candidates. BP assignment is based on a structural formula of the targeted compound. Thus fragments are also predicted as structural formulae.

For each BP, two types of bond cleavage, “direct” and “rearranged,” are computed. When a BP is cleaved, the heteroatom adjacent to the bond is assumed to be protonated. Since our study presupposes fragmentation in positive-ion mode ESI-MS/MS, only positive even-electron ions are considered.^13)^ Direct cleavage does not accompany hydrogen movement; a positive charge moves to the carbon atom. Rearranged cleavage is associated with hydrogen movement from the carbon atom side to the heteroatom. If small molecules such as water, ammonia, ethylene, and carbon monoxide are dissociated, ionization of such small molecules is ignored.

**Figure 1:**
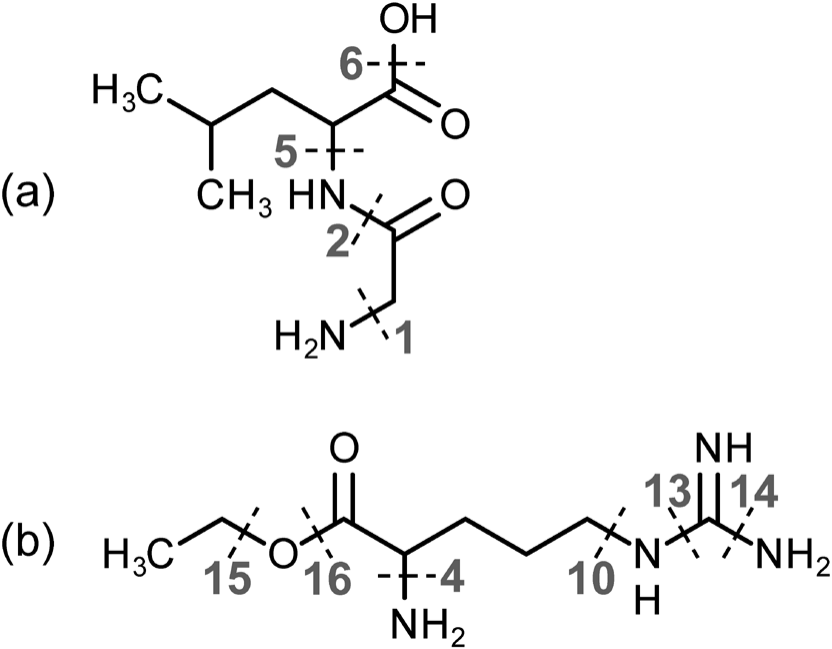
Fig. 1. Assignment of BPs on (a) glycylleucine and (b) ethyl argininate. Bonds crossed by dashed lines are assigned BPs.

All BPs analyzed in the present study are summarized in Table 1. They are chosen from two example molecules: glycylleucine and ethyl argininate (Fig. 1 (a) and (b), respectively). The numbers in Fig. 1 correspond to the number of BPs described in Table 1. Cleavage activation energy for each BP is an indicator of cleavage tendency. By assigning BPs and their cleavage activation energy on chemical bonds composing a metabolite structure, fragmentation pathways constituted by several bond cleavage steps are predicted.

**Table 1:** Table 1. Bonding patterns analyzed in the present study.

No.	Bound atoms	Neighboring groups		Activation energy (kJ mol^−1^)	
				Direct	Rearranged
1	C–N	C(–CONH_2_)	N(none)	203	—
2	C–N	C(–CH_3_, =O)	N(–CH_3_)	124	95
3	C–N	C(–CH_3_, =O)	N(=CH_2_)	100	68
4	C–N	C(–CH_3_, –COOH)	N(none)	236	—
5	C–N	C(–CH_3_, –COOH)	N(–COCH_3_)	197	161
6	C–O	C(–CH_3_, =O)	O(none)	25	—
7	C–C	C(–NH_2_)	C(=O)	−99	—
8	C–C	C(–CH_3_, –NHCH_3_)	C(=O)	−153	—
9	C–N	C(–CH_3_)	N(none)	245	—
10	C–N	C(–CH_3_)	N(–CNHNH_2_)	238	132
11	C–N	C(–CH_3_)	N(=C=NH)	206	—
12	C–N	C(=NH)	N(–CH_3_)	403	—
13	C–N	C(–NH_2_, =NH)	N(–CH_3_)	105	79
14	C–N	C(–NHCH_3_, =NH)	N(none)	51	—
15	C–O	C(–CH_3_)	O(–COCH_3_)	—	105
16	C–O	C(–CH_3_, =O)	O(–CH_3_)	28	91
17	C–C	C(–CH_3_, –NH_2_)	C(=O)	−129	—

To make the computation simpler, substructures distant from the bond are replaced with hydrogen atoms. For example, in BP 5, a precise group next to the bond is isobutyl (–CH_2_CH(CH_3_)_2_). However, the group is replaced with a methyl group (–CH_3,_ see Fig. 1 (a) and Table 1). This simplification is applied for all BPs.

### Calculation of Activation Energy

Activation energy required to cleave each BP is calculated with computational chemistry software. First, a stereochemical model of each BP is built computationally. Next, a reaction pathway from the initial structure to a cleaved state is simulated, and the corresponding energy difference along the dissociation is calculated by Reaction plus software.^15)^ If the dissociation process exhibits a maximal energy between the initial and the cleaved state, transition state optimization starting from the structure with a maximal energy is performed with Gaussian 09.^16)^ Here, activation energy is defined as the energy difference between the initial state and the transition state. If the initial or cleaved state has the maximal energy, activation energy is defined as the energy difference from the initial state to the cleaved state. This means that the activation energy is negative when the initial state is high-energy.

High-precision Gibbs free energy of the initial, transition, and fragmented state (ions and neutrals) is calculated with the CBS-QB3 method,^17)^ where a typical error is around 1 kcal mol^−1^.^18)^ Structural optimization of all molecules is performed with the density functional theory (DFT) method. Functional and basis set are CAM-B3LYP including the long-range correction and 6−31+G(d,p) including polarization and diffusion of atomic orbital, respectively. The long-range correction is important for bond-breaking and making reactions.^19)^ All calculations here are done with Gaussian 09.

Energy calculation of BPs considers three-dimensional structure, but the assignment of BPs to structures does not consider stereochemistry in our method.

### Computational Time

The computation time depends on the structural environment, especially the number of atoms, of each BP. It typically takes around three hours to calculate activation energy of one BP, which includes reaction pathway optimization and high-precision energy calculation. In a few cases, computational time exceeded ten hours. For example, computation for BP 5 composed of 18 atoms required 19 h to obtain activation energy.

Computational environments are as follows. For reaction pathway optimization, 246.4 GB memory (7.7 GB×32 cores, Intel Xeon E5-2690) was utilized. For structural optimization and high-precision energy calculation of each molecule (initial, transition, and fragmented states), 61.6 GB memory (7.7 GB×8 cores, Intel Xeon E5-2690) was utilized. All the computations were executed with supercomputer systems in Research Center for Computational Science, Okazaki, Japan.

## RESULTS

### Strategy of the Prediction Using Bonding Patterns

Our fragment pathway prediction is based on assignment of BPs, which represent the vicinity of cleaved bond, with their cleavage activation energy. All bonds composing a metabolite are scanned, and candidates for bond cleavage, *i.e.*, single bonds formed by a carbon atom and a heteroatom, are transcribed as BPs which include two atoms forming the bond and its neighboring chemical groups. A list of activation energy of BPs is referred to, and values of activation energy are assigned on the candidate bonds to be cleaved. By cleaving the candidate bonds with low activation energy, formal fragment structures are predicted. Assignment of BPs on the predicted structural formulae of fragments predicts their further fragmentation. Tracing the low-energy fragmentation pathways yields a set of fragments likely to be observed in measurements.

We have predicted fragment structures of several metabolites with the energetic computation on bond cleavage. To annotate structural formulae to fragment peaks in experimental standard product ion spectra of reserpine, 2′-deoxycytidine 5′-phosphate, 3-indoxyl sulfate, phosphocholine, isoproturon, and kaempferide, we predicted their fragmentation pathways to confirm that bond cleavage energy along the pathways were low enough.^12)^ Energetic analysis on fragmentation of a hydroxyl ceramide elucidated a true position of a hydroxy group of the ceramide and therefore β-hydroxyl ceramide was identified from a biological sample without experimental standard spectra.^14)^ Here, we will present example processes of fragment prediction on glycylleucine and ethyl argininate.

Activation energy for cleavage of each BP can be calculated irrespective of metabolite structures and stored in the form of Table 1, though here only BPs included in glycylleucine and ethyl argininate are listed to explain examples of the prediction. By expanding the table of calculated activation energy for BPs, the coverage of our prediction method becomes broader. Rough estimation of the number of BPs possibly existing in all metabolites is presented in DISCUSSION section. How to reduce the BPs in order to cover the huge metabolite space is also discussed.

### Glycylleucine

**Figure 2:**
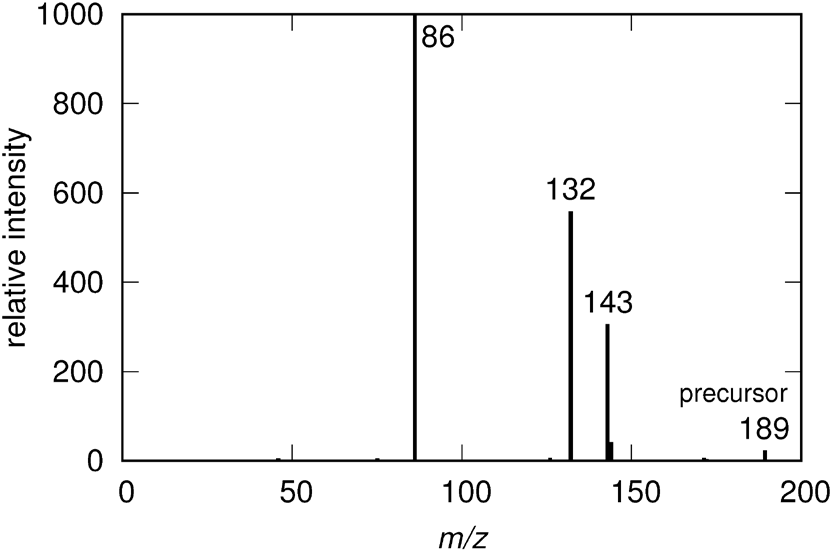
Fig. 2. A standard product ion spectrum of glycylleucine (MassBank ID: KO003025). Numerals on top of major peaks indicate *m*/*z* values. The spectrum was experimentally obtained by positive ion mode LC-ESI-QQ MS/MS with collision energy=20 eV.

A standard product ion spectrum of glycylleucine from MassBank database (MassBank ID: KO003025)^2)^ is shown in Fig. 2. Major product ions contain *m*/*z* 86, 132, and 143. We predicted product ions of glycylleucine and compared the prediction result with the standard spectrum.

Activation energy for cleavage of each BP in glycylleucine was assigned from the upper part of Table 1. The lowest activation energy was obtained as direct cleavage of BP 6 (25 kJ mol^−1^), *i.e.*, the C–O bond was the easiest to cleave. This cleavage corresponded to water elimination from a carboxyl group, which is common fragmentation in MS/MS.^9)^ Rearranged cleavage of BP 2 showed relatively low activation energy (95 kJ mol^−1^). This was an amide cleavage and its product ion was equivalent to the *y*-type ion of a general peptide bond cleavage.^20)^ Through this analysis, we could confirm that the activation energy was indeed lower for experimentally obtained fragmentation.

**Figure 3:**
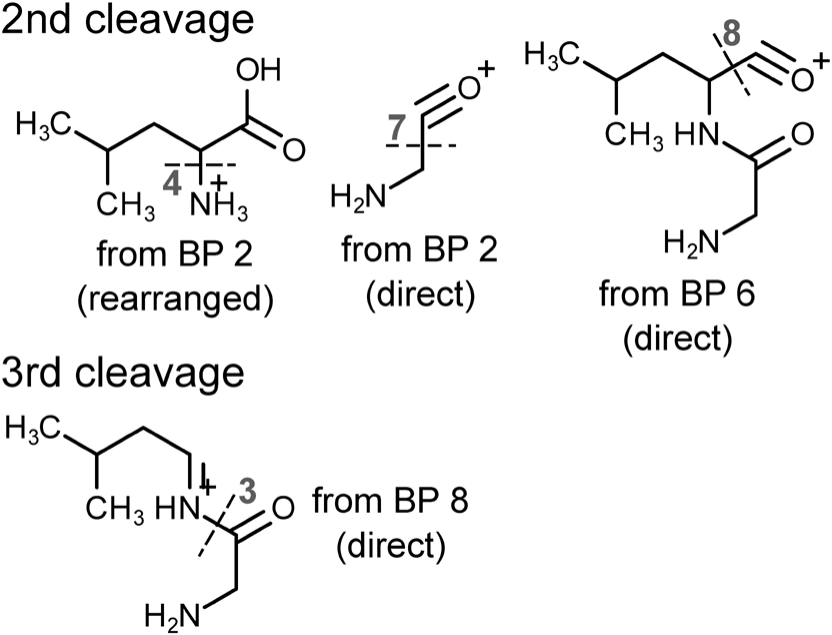
Fig. 3. Second and third fragmentation of product ions from glycylleucine. The numerals correspond to the number of BPs in Table 1. Cleavage of the BP leading to each fragment is shown on its bottom or right.

Next, activation energy for further fragmentation of glycylleucine was assigned (Fig. 3 and Table 1). In the second and third fragmentations, C–C bonds might become cleavage candidates because a carbon atom might be charged after the first fragmentation. A positively charged carbonyl group resulting from the cleavage of BP 2(direct) or 6(direct) may be dissociated as a carbon monoxide. In this case, the C–C bond cleavage becomes energetically favorable; otherwise the C–C cleavage requires much higher energy and not regarded as a candidate for fragmentation.

Some of the second fragmentations showed negative activation energies, *i.e.*, the product structure is more stable than the initial structure. In particular, cleavage of BP 6 is followed by the cleavage of BP 8 immediately (activation energy=−153 kJ mol^−1^) and the product ions may undergo further rearranged cleavage of BP 3 with relatively low activation energy (68 kJ mol^−1^). In the standard spectrum of glycylleucine in Fig. 2, however, *m*/*z* 171 has a slight peak whose intensity is 0.6% of *m*/*z* 86. That means the product ion of *m*/*z* 171 can remain. The direct cleavage of BP 6 causes a *b*-type ion of the dipeptide and it is known to be stabilized by cyclization.^21)^ In a standard spectrum obtained with collision energy (CE) 10 eV (MassBank ID: KO003024), *m*/*z* 171 becomes more intense. This fact implies that the cyclization occurs; with lower CE, lifetime of a product ion becomes longer and probability of the cyclization becomes greater. This is an example of inconsistency between a formal structure obtained by simple bond cleavage and a real one including recombination. Although it is out of our scope, the possible inconsistency should be noted.

**Figure 4:**
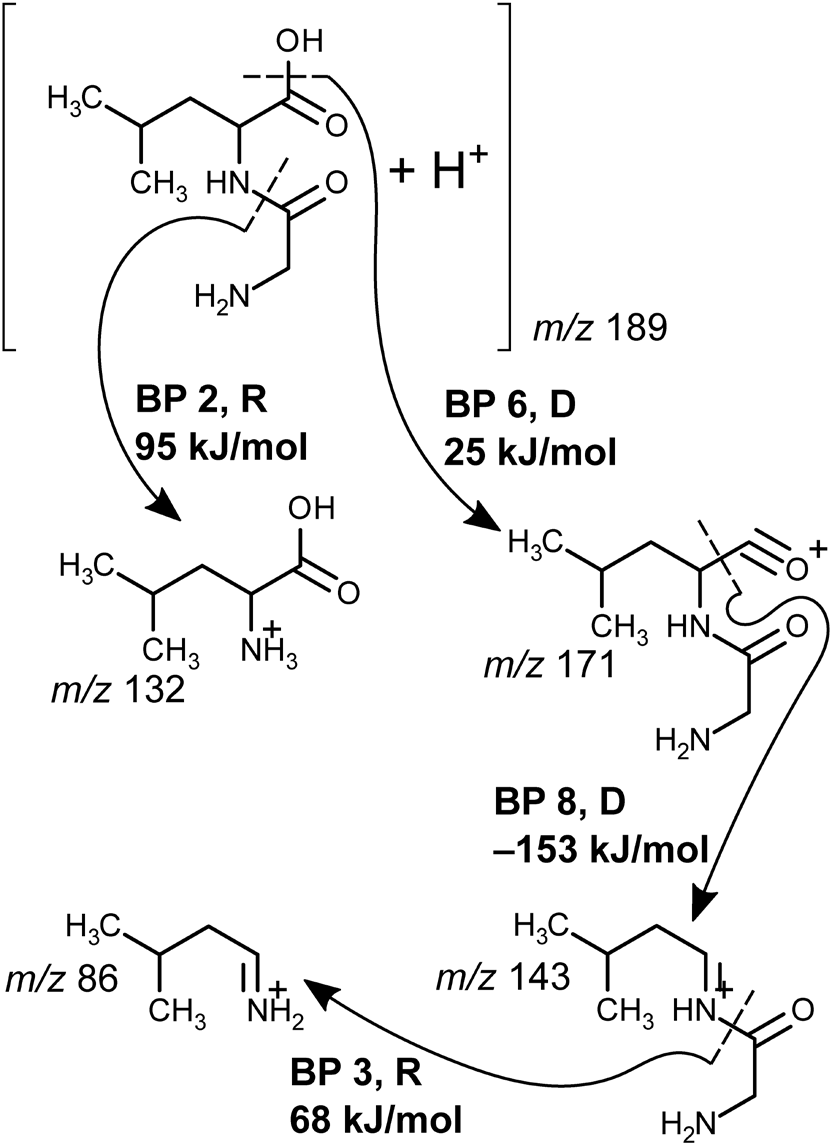
Fig. 4. Fragmentation pathways of glycylleucine predicted with activation energies. The cleaved BP with D (direct) or R (rearranged) and its activation energy are described. Initial direction of arrows indicates the side of the protonated substructures.

Fragmentation pathways of glycylleucine were predicted from the activation energies. The pathway with the lowest energy, *i.e.*, the most likely to occur in experiments, was the consecutive cleavage of BPs 6(direct)→8(direct)→3(rearranged), where activation energies were 25→−153→68 kJ mol^−1^, respectively. Rearranged cleavage of BP 2 (activation energy=95 kJ mol^−1^) was also energetically favorable, but the cleavage would not proceed to the next cleavage of BP 4 due to its high activation energy (253 kJ mol^−1^). The predicted fragmentation pathways are illustrated in Fig. 4. Compared with a measured standard spectrum from MassBank database (MassBank ID: KO003025)^2)^ in Fig. 2, our prediction successfully reproduced the major fragmentations with CE=20 eV.

The fragment of *m*/*z* 132 is identical to a protonated molecule of leucine. In a measured product ion spectrum of leucine (MassBank ID: KO003279), a fragment of *m*/*z* 115 is observed. It results from direct cleavage of BP 4 whose activation energy is high (236 kJ mol^−1^). A product ion spectrum of glycylleucine with higher CE of 50 eV (MassBank ID: KO003028) indeed contains fragments of *m*/*z* 43, 44, and 113 of large intensities, requiring cleavage of carbon–carbon bonds. Such cleavage is not considered in the present study because of their high activation energy. This apparent inconsistency is due to our ignorance of chemical kinetics. Competition between high- and low-energy fragmentation pathways and dependence of fragmentation on provided CE rely on reaction rates. The consideration of reaction rates is an important future work of our method.

### Ethyl Argininate

**Figure 5:**
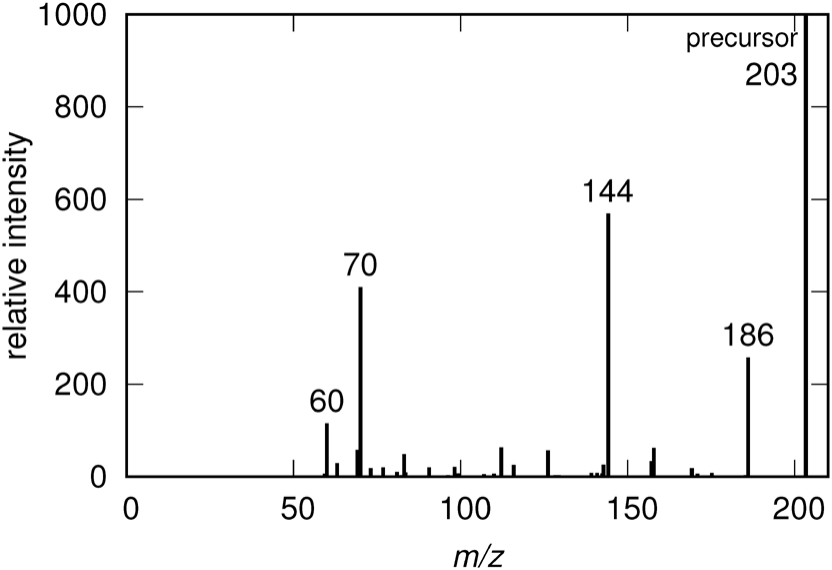
Fig. 5. A standard product ion spectrum of ethyl argininate (MassBank ID: KO002249). Numerals on top of major peaks indicate *m*/*z* values. The spectrum was obtained by positive ion mode LC-ESI-QQ MS/MS with collision energy=20 eV.

A standard product ion spectrum of ethyl argininate from MassBank database (MassBank ID: KO002249)^2)^ is shown in Fig. 5. Major peaks are on *m*/*z* 60, 70, 144, and 186. We also predicted product ions of ethyl argininate and compared the prediction result with the standard spectrum.

Activation energy for cleavage of each BP in ethylargininate was assigned from the lower part of Table 1 (see Fig. 1 (b)). Here, BP 4, which appeared in the glycylleucine molecule, was assigned again on the middle amino group. The lowest energy was calculated as the direct cleavage of BP 15 (28 kJ mol^−1^), which corresponded to the dissociation of an alcohol from the ester bond moiety. It is noteworthy that this cleavage is similar to water elimination from the carboxyl group with the lowest activation energy (25 kJ mol^−1^) in BPs on glycylleucine. This fact implies that effects on bond cleavage of a methoxy group is not much different from that of a hydroxy group. Rearranged cleavage of BP 13 (dissociation of carbodiimide from the guanidine moiety) and direct cleavage of BP 14 (ammonia elimination) also showed relatively low activation energy (79 and 51 kJ mol^−1^, respectively).

**Figure 6:**
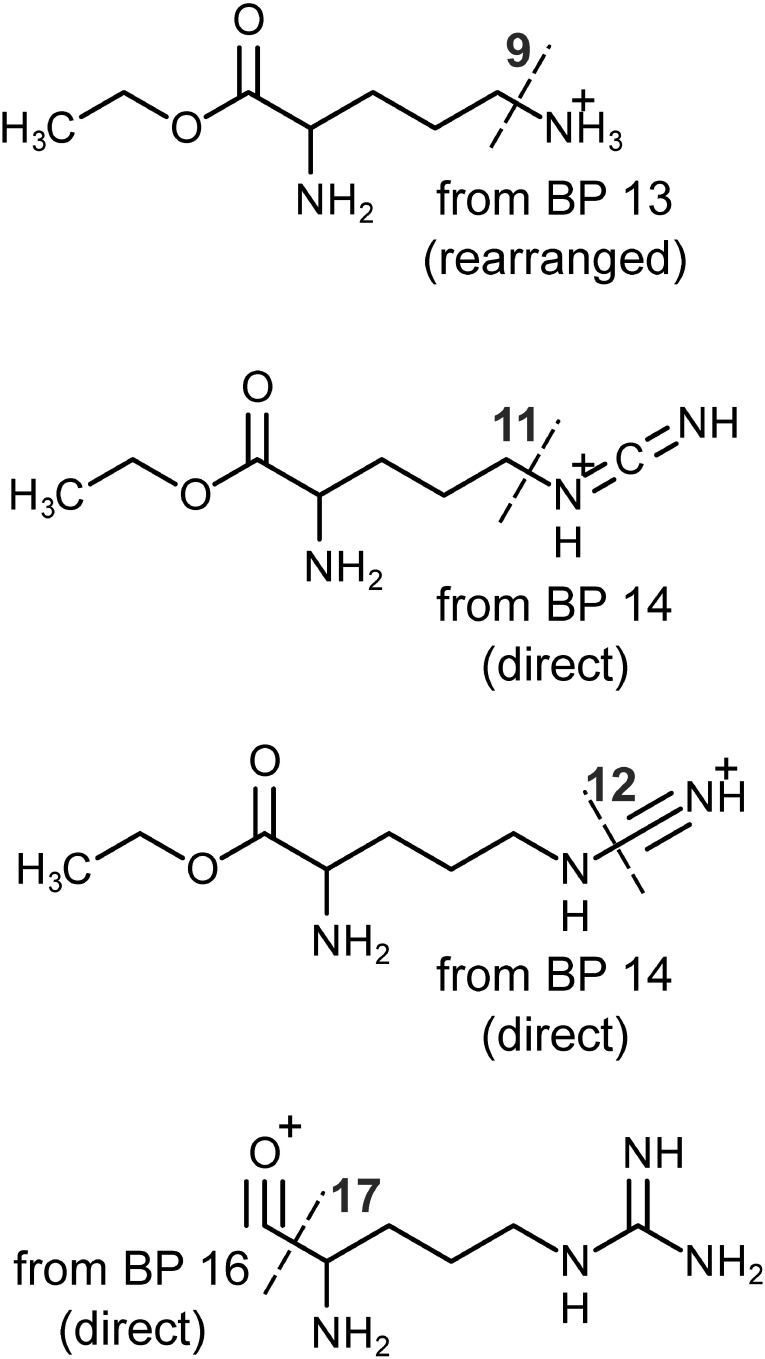
Fig. 6. Second fragmentation of product ions from ethyl argininate. Two fragmentation pathways can be considered for resonance structures of the product ion from direct cleavage of BP 14.

For ethyl argininate, no third-step cleavage was considered stable and only second-step cleavage was investigated (see Fig. 6). The fragmentation pathway with the lowest activation energy was the cleavage of BPs 16(direct)→17(direct) (28→−129 kJ mol^−1^, respectively), which resulted in the fragment of *m*/*z* 129. The other second fragmentation required more than 200 kJ mol^−1^ to be activated.

**Figure 7:**
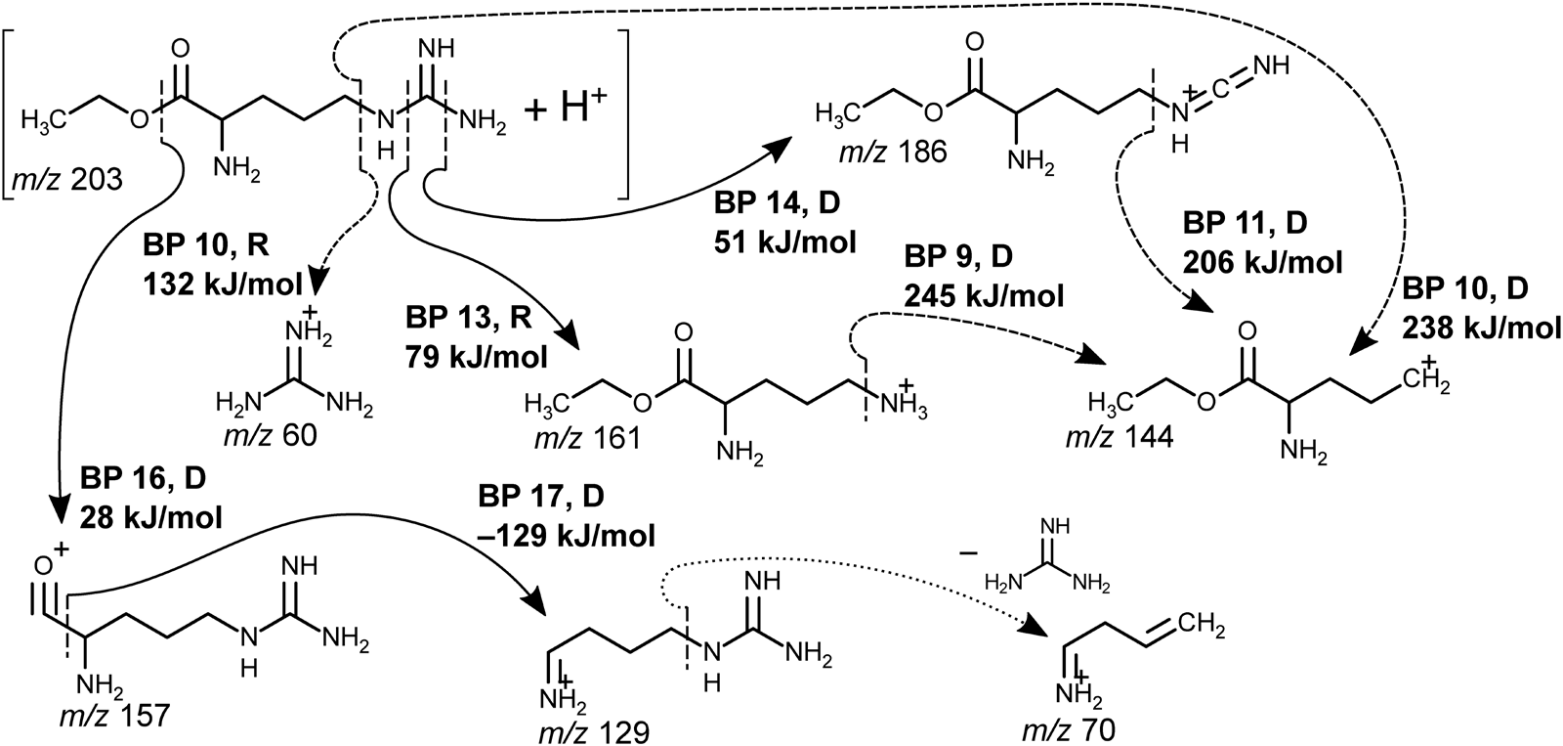
Fig. 7. Tentative fragmentation pathways of ethyl argininate. Solid and dashed arrows indicate pathways with low activation energy and high activation energy (>100 kJ mol^−1^), respectively. The cleaved BP with D (direct) or R (rearranged) and its activation energy are described. The pathway with a dotted arrow is not predicted but explaining the standard spectrum. Initial direction of the arrows indicates which side of the cleaved bond is detected as a product ion.

By selecting relatively low activation energy from the calculation results, *m*/*z* values of fragments predicted to be produced are 161 (rearranged cleavage of BP 13 activated by 79 kJ mol^−1^), 186 (direct cleavage of BP 14 activated by 51 kJ mol^−1^), 157 (direct cleavage of BP 16 activated by 28 kJ mol^−1^), and 129 (consecutive direct cleavage of BPs 16 and 17 activated by 28 and −129 kJ mol^−1^, respectively) (see Fig. 7). Rearranged cleavage of BP 16 can be activated by relatively low 91 kJ mol^−1^ of energy; whereas, direct cleavage of BP 16 is activated by much lower energy (28 kJ mol^−1^) and may suppress the rearranged cleavage of BP 16 as its competitor. This prediction was coincided with a measured standard spectrum in Fig. 5 only at one major peak on *m*/*z* 186.

Two of the major peaks (*m*/*z* 144 and 60) in the standard spectrum were predicted to require high activation energy (238 kJ mol^−1^ for direct cleavage of BP 10 and 132 kJ mol^−1^ for rearranged cleavage of BP 10, respectively). The fragment with *m*/*z* 144 could also be produced from two-step cleavage of BPs 14(direct)→11(direct), which required 51→206 kJ mol^−1^, respectively, or BPs 13(rearranged)→9(direct), which required 79→245 kJ mol^−1^, respectively. The former was the lower-energy pathway to produce the fragment of *m*/*z* 144 than the latter; however, the maximum activation energy of 206 kJ mol^−1^ is still high. On ethyl argininate, intensities of the product ions relative to the precursor ion were lower in comparison with glycylleucine. This implies that the precursor ion of ethyl argininate is harder to decompose than that of glycylleucine. This is consistent with the observed relatively high activation energies. In a CID study on protonated arginine,^22)^ a product ion losing ammonia corresponding to direct cleavage of BP 14 was observed, while loss of carbodiimide corresponding to the rearranged cleavage of BP 13 was not observed. Forbes and coworkers^23)^ confirmed that an ammonia-dissociated product ion of protonated arginine can lose carbodiimide (a production of *m*/*z* 144). This observation supports that the fragment of *m*/*z* 144 is produced from the two-step cleavage of BPs 14 (direct)→11 (direct).

The fragment of *m*/*z* 70 never appeared in our prediction. This missing fragment most likely came from a charge remote fragmentation (CRF) (see Fig. 7). It is produced when the fragment produced with the lowest activation energy, whose *m*/*z* is 129, losing 59 mass. That loss of mass is regarded as elimination of guanidine moiety on the right side of ethyl argininate. In the present prediction, no CRF was considered. Energy released by the cleavage of BP 17 (129 kJ mol^−1^) might help the further fragmentation. Glycylleucine also showed an example of missing CRF. Protonated leucine is caused by the rearranged cleavage of BP 2. Generally, protonated amino acids lose mass of 46 corresponding to HCOOH or H_2_O+CO. However, as the cleavage is not mediated by the charged amide nitrogen, the loss of 46 mass does not appear in our prediction (see Fig. 4).

Another inconsistency was the absence of *m*/*z* 161 in the standard spectrum despite its relatively low activation energy (79 kJ mol^−1^). This is explained by further loss of ammonia resulting in *m*/*z* 144; however, activation energy of the cleavage is high (245 kJ mol^−1^). Such high activation energy should come from an unstable structure of the fragment ion having a charge on the carbon atom connected by two hydrogen atoms. The highly electrophilic structure could absorb electron lone pairs on the amino group or the carbonyl group to cause a ring structure, and the rearrangement could accelerate the ammonia elimination. In the introduction of our BPs, remote structures were ignored and caused the discrepancy between computation and actual fragmentation.

Csonka and coworkers^24)^ investigated pathways for guanidine and ammonia losses from protonated arginine. They calculated the energy barrier for *S*_N_2 attack of the α-amino group to eliminate guanidine. It was about 100 kJ mol^−1^ lower than the activation energy of direct cleavage of BP 10. The intense *m*/*z* 144 fragment can be produced *via* the nucleophilic attack, and the *m*/*z* 70 fragment can be explained with the nucleophilic attack: elimination of ethyl formate (or ethanol and carbon monoxide) from the product ion caused by the *S*_N_2 elimination of guanidine results in the fragment of *m*/*z* 70. Recombination of a remote structure may play an important role in fragmentation, but our prediction ignores such remote effect. This discrepancy becomes remarkable when a target molecule has a long chain structure like arginine. It is known that cyclization in a charged peptide prior to its fragmentation enhances bond cleavage.^25)^ The consideration of such cyclization is one of our future works.

## DISCUSSION

To apply our prediction to any metabolites, we need to include all BPs which appear in metabolites. The question is how many BPs exist in metabolite structures. When considering nearest-neighbor four kinds of atoms (hydrogen, carbon, nitrogen, and oxygen) to a cleaved bond, the number of BPs becomes as follows: 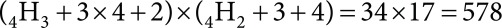
(1)where H is “repeated combination,” which is computed as *_n_*H*_r_=_n_*_+_*_r_*_−1_ C*_r_*. The terms in Eq. (1) correspond to the structures described in Eq. (2). 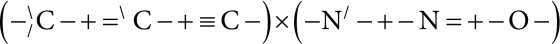
(2)


The first parentheses in Eq. (2) illustrate the patterns of the carbon atom side: a carbon atom can make three single bonds (_{H_*_,_*_C_*_,_*_N_*_,_*_O}_H_3_), one double bond and one single bond ({C, N, O}×{H, C, N, O}), or one triple bond ({C, N}). The second parentheses in Eq. (2) illustrate the patterns of the heteroatom side: a nitrogen atom can make two single bonds (_{H_*_,_*_C_*_,_*_N_*_,_*_O}_H_2_) or one double bond ({C, N, O}), and an oxygen atom can make one single bond ({H, C, N, O}). If the patterns are extended to a broader range which is reached by two bonds from the cleaved bond, the number of patterns becomes roughly 70,000.

We are considering some strategies to reduce the number of BPs. First of all, the estimation above includes unnatural patterns like a nitrogen atom connected by two nitrogen atoms. Such patterns can be excluded by searching metabolite database.

Superposition of effects from multiple chemical groups should also be investigated. If an effect from a set of three methyl groups can be represented by superposing an effect from one methyl group, the number of BPs decreases drastically. In the fragment prediction of glycylleucine and ethyl argininate, there were some sets of similar BPs. For example, BPs 6 and 16 were different only about the oxygen atom connected by a hydrogen atom and a methyl group, respectively, and their activation energies were almost same. BPs 7, 8, and 17 correspond to elimination of carbon monoxide. With more methyl groups added, their activation energies became lower (BP 7>17>8, see Table 1). These results suggest that addition of methyl groups on a carbon atom side reduces activation energy in contrast to addition on an oxygen atom side. Additional calculation of BPs with more methyl groups will contribute to elucidate superposed effects from multiple chemical groups.

Comparison of similar chemical groups is also important to simplify structures included in BPs. BP 9 is the most basic one, which includes only a methyl group as its neighboring group. BP 1 has a neighboring group founded on a carbon atom (–CONH_2_), but the group is different from a methyl group and lowers activation energy by 42 kJ mol^−1^. On the other hand, BP 4 including a methyl group and a carboxyl group, which are founded on carbon atoms, has similar activation energy to the basic BP 1. Such comparison will clarify whether chemical groups on a carbon atom, like –CONH_2_ and a carboxyl group, can be treated as a methyl group or not. We are now investigating it by computing BPs composed of several carbon modifications.

## CONCLUSION

Current standard product ion spectral library is not sufficient for comprehensive identification of metabolites. We have presented fragmentation pathway prediction by means of computational quantum chemistry, and the prediction well matched with the standard spectrum of a dipeptide, glycylleucine. For another molecule, ethyl argininate, additional fragmentation pathways with high activation energy and recombination were required to reproduce its standard spectrum.

The examples show benefits and limitation of the approach: assignment of bonding patterns (BPs) and activation energy calculation. By focusing on a local structure, BPs are independent of size of metabolites and can be applied widely to most metabolites. Energetical analyses can provide quantitative results on fragmentation mechanism. However, two improvements are required to extend applicability of the prediction method. One is the introduction of charge remote fragmentation, which can be implemented by assigning BPs on bonds without ionization. The other is the consideration of energetic dependency applied to accelerate fragmentation in experimental systems. Activation energy can be converted into reaction rate of the fragmentation and its dependency on applied energy or temperature can be computed with chemical kinetics theory. The computation, however, will become combinatorially complex. From quantitatively detailed energetics, we plan to estimate plausible threshold of activation energy to judge whether a fragment ion is produced.

To extend the coverage of our prediction, we need to calculate more BPs and store their activation energy. As a simple estimation suggests enormous number of BPs to be calculated, we need to decrease the number by merging chemical groups having similar energetical effects. To achieve that, we currently work on additional computations in order to obtain correct simplification of chemical groups included in BPs.

## Notes

Mass Spectrom (Tokyo) 2018; 7(1): A0066
